# Comparison of Chemotherapy Combined With Chidamide Versus Chemotherapy in the Frontline Treatment for Peripheral T-Cell Lymphoma

**DOI:** 10.3389/fimmu.2022.835103

**Published:** 2022-02-02

**Authors:** Jinni Wang, Ning Su, Yu Fang, Shuyun Ma, Yuchen Zhang, Jun Cai, Qihua Zou, Xiaopeng Tian, Yi Xia, Panpan Liu, Zhiming Li, He Huang, Huiqiang Huang, Qingqing Cai

**Affiliations:** ^1^State Key Laboratory of Oncology in South China, Collaborative Innovation Center of Cancer Medicine, Sun Yat-sen University Cancer Center, Guangzhou, China; ^2^Department of Medical Oncology, Sun Yat-sen University Cancer Center, Guangzhou, China; ^3^Department of Oncology, Guangzhou Chest Hospital, Guangzhou, China

**Keywords:** peripheral T-cell lymphoma, HDAC inhibitor, chidamide, chemotherapy, frontline

## Abstract

**Background:**

Peripheral T-cell lymphoma (PTCL) is featured with a poor survival outcome. China has approved chidamide, an oral novel histone deacetylase inhibitor, for patients diagnosed with relapsed or refractory PTCL.

**Objective:**

We compared the benefit of traditional chemotherapy alone and a combination of chidamide and traditional chemotherapy against newly diagnosed PTCL. Prognostic factors related to progression and survival in patients diagnosed with untreated PTCL were also investigated.

**Methods:**

104 patients with newly diagnosed PTCL were enrolled and divided into chemotherapy (ChT) group and chemotherapy combined with chidamide (ChT+C) group. Survival curves were plotted by the Kaplan-Meier method. Univariate and multivariate analysis were conducted with Log-rank test and Cox’s proportional hazard regression. Subgroup analysis and interaction tests were conducted to evaluate factors associated with prognostic differences between ChT and ChT+C groups.

**Results:**

Compared with patients in ChT group, those in ChT+C group had superior progression-free survival (PFS) (*p*=0.047). However, there was no significantly statistical difference observed between the two groups in overall survival (OS) (*p*=0.212). High IPI scores have a negative relationship with survival. Multivariate analysis revealed that the type of frontline treatment regimen is an independent factor associated with PFS of PTCL patients (*p*=0.045). In the subgroup of patients with high international prognostic index scores (3-5), the HR value for PFS comparing ChT with ChT+C was 4.675. A test of interaction between IPI and treatment showed statistical significance (*p* = 0.037), implying that the benefits of ChT+C are higher for patients with high IPI scores.

**Conclusions:**

In summary, the combination of ChT and chidamide may provide a promising prospect for patients with newly diagnosed PTCL.

## Introduction

Peripheral T-cell lymphomas (PTCL) comprise a group of rare lymphoid malignancies with distinct phenotypes and clinical presentations (1). In Western countries, these aggressive lymphomas account for 10–12% of non-Hodgkin lymphomas (NHL), but in Eastern Asian for 20% (2, 3).

Frontline treatment of PTCL is mainly supplied from the experience in treatment of B-cell counterparts. Unfortunately, compared with B-cell NHL, PTCLs have a poorer prognosis under conventional treatment. Except for anaplastic lymphoma kinase (ALK)–positive anaplastic large cell lymphoma (ALCL), complete and durable response rates are disappointingly low with most commonly employed anthracycline-based regimens (4, 5). Even though the intensive-dose induction therapy consolidated with autologous stem-cell transplantation (ASCT) in first remission was applied, 18% patients with PTCL still suffered from disease relapses and progressions within 2 years (6). The ECHELON-2 trial displayed the efficacy and safety of brentuximab vedotin in the frontline setting, but only for CD30-positive peripheral T-cell lymphomas (7). Thus, there is an urgent practical necessity for innovative treatment strategies that are highly effective and tolerable for patients with PTCL.

Globally, HDAC inhibitors (HDACi) belinostat, romidepsin and chidamide, with the overall response rate (ORR) ranging from 25% to 28%, have been approved for relapsed or refractory PTCL (8–10). These inhibitors promote differentiation, growth arrest, and apoptosis in neoplastic cells *in vitro* (11). The efficacy of HDACi may be attributed to the epigenetic dysregulation in PTCL (12). China has approved chidamide, an oral novel histone deacetylase inhibitor, for patients with relapsed or refractory PTCL, based on a small phase II trial showing a high ORR of 28%. However, there is still a lack of consensus on the application of chidamide in the frontline setting. Furthermore, with modest effectiveness, HDACi monotherapy is not capable to completely overcome the poor prognosis of PTCL. Thus, the optimal therapeutic approach for patients with PTCL need to be further investigated.

In the present research, we compared the efficacy of traditional chemotherapy alone and traditional chemotherapy combined with chidamide against newly diagnosed PTCL. Prognostic factors having an impact on progression and survival of patients diagnosed with untreated PTCL were also explored.

## Materials and Methods

### Patients

**Figure 1:**
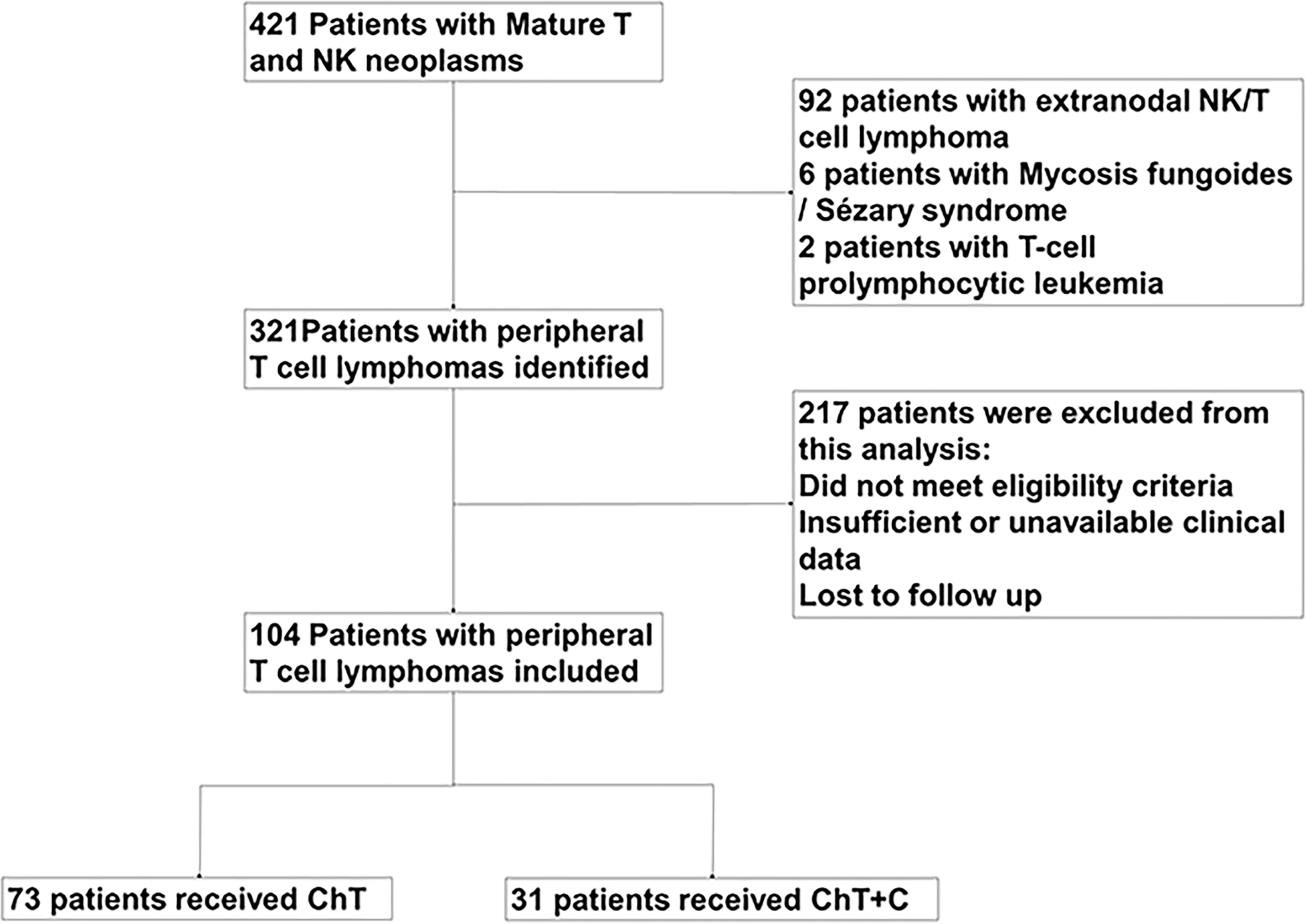
The process of patients inclusion.

421 PTCL patients managed in Sun Yat-sen University Cancer Center from January 2014 to July 2020 were reviewed, in which 104 patients were diagnosed with newly diagnosed PTCL and retrospectively analyzed in our study, as shown in **Figure 1**. Except for T-cell prolymphocytic leukemia, Sézary syndrome, mycosis fungoides and extranodal NK/T cell lymphoma, nasal type, other subtypes of untreated PTCL patients were enrolled. This study was approved by the institutional ethical review board of Sun Yat-sen University Cancer Center.

### Study Design

Our study retrospectively analyzed the efficacy of chemotherapy (ChT) and chemotherapy +chidamide (ChT+C) among patients with newly diagnosed PTCL. According to the frontline treatment they received, patients were divided into two treatment groups, one was ChT group, the other one was ChT+C group. The primary endpoint was progression-free survival (PFS). The overall survival (OS), objective response rate (ORR) and complete remission (CR) rate were secondary endpoints. PFS was calculated from the initiation of frontline treatment to the time point of relapse, progression, start of second-line treatment, death, or the last follow-up. OS was estimated as the time period from diagnosis to death or last follow-up. EBER *in situ* hybridization was applied to detection EBV infection. Computed tomography, magnetic resonance imaging and positron emission computed tomography were repeated every two or three cycles in order to evaluate the disease response, which was performed according to 2016 Lugano Classification lymphoma response criteria. The toxicity grading was conducted based on the National Cancer Institute Common Toxicity Criteria.

### Statistical Analysis

The differences in clinicopathological characteristics between the two groups were analyzed by χ2 test or Fisher’ s exact test. Survival curves were generated using the Kaplan-Meier method. A univariate analysis of survival was conducted using the log-rank test. The variables included for the univariate analyses were as follows: treatment group, International Prognosis Index (IPI), age, Ann Arbor stage, serum lactic dehydrogenase (LDH), Eastern Cooperative Oncology Group (ECOG) performance status, extranodal sites and EBER. Multivariate Cox’s regression analysis was performed for significant variables identified by using univariate analysis to determine independent prognostic factors.

To further evaluate factors associated with prognostic differences between ChT and ChT+C group, subgroup analysis was performed to assess survival outcomes of treatment groups in subgroups defined according to IPI (high scores of 0–2 and low scores of 3-5), LDH (normal and elevated), age (≤60 and >60 years), stage (I-II and III-IV), gender (male and female) and EBER (positive and negative). Interaction tests were performed on the patients enrolled in our study. Results are presented using forest plot with *p* values for the interaction effects and hazard ratios.

Since baseline imbalance and chemotherapy regimen may generate discrepancies in treatment outcome between groups, we conducted a 1:1 matched case-control analysis with the propensity score matching method. A total of 16 patients treated with CHOP-C and 16 patients treated with CHOP were included in further analysis. In the matched groups, IPI were matched between groups. Based on the matched population, survival analysis was conducted using the Kaplan–Meier method.

Statistical analysis was conducted with IBM SPSS Statistics 20.0 software. A p-value less than 0.05 was considered statistically significant.

## Results

### Baseline Characteristics

Included in our analysis were 104 patients with newly diagnosed PTCL. The baseline characteristics of the patients was shown in **Table 1**. The median age was 59.5 years (range 24–87 years). Of the 104 patients, 37 were males (35.6%), whereas 67 (64.4%) were females. The ChT group comprised 73, whereas 31 patients were included in the ChT+C group. The mean IPI in both groups was 2. Compared with ChT+C group, the ChT group comprised of more PTCL-NOS (54.8% vs 22.6%), and less AITL (13.7% vs 51.6%) patients. The two treatment groups did not differ in other characteristics. A total of 52 (71.2%) and 17 (54.8%) in ChT group and ChT+C group, respectively, received doxorubicin-based regimens as frontline chemotherapy. Meanwhile, 9 (12.3%) and 12 (38.7%) in ChT group and ChT+ C group, respectively, received etoposide-based regimens as frontline chemotherapy. Only two of patients included in this study underwent ASCT in the frontline setting. 17 patients received maintenance treatment with HDACi. In ChT group, 33 patients had available salvage treatment information, including 20 patients receiving chemotherapy, 7 patients receiving chidamide-contained treatment, 4 patients enrolled in clinical trials and 2 patients receiving other targeted treatment. In ChT+C group, 6 patients had available salvage treatment information, including 2 patients receiving chemotherapy, 2 patients receiving chidamide-contained treatment, 1 patient receiving lenalidomide and 1 patient receiving radiotherapy.

**Table 1:** Baseline characteristics of 104 PTCL patients.

Characteristics	Overall n = 104	ChT n = 73	Chidamide+ChT n = 31	*p*
Age, years				0.562
median	59.5	59	60	
range	(24-87)	(24-87)	(27-84)	
Gender				0.377
Male	37 (35.6%)	24 (32.9%)	13 (41.9%)	
Female	67 (64.4%)	49 (67.1%)	18 (58.1%)	
LDH				0.084
Normal	57 (54.8%)	36 (49.3%)	21 (67.7%)	
Elevated	47 (45.2%)	37 (50.7%)	10 (32.3%)	
ECOG				0.103
0-1	92 (88.5%)	62 (84.9%)	30 (96.8%)	
≥2	12 (11.5%)	11 (15.1%)	1 (3.2%)	
Stage				0.770
I-II	22 (21.2%)	16 (21.9%)	6 (19.4%)	
III-IV	82 (78.8%)	57 (78.1%)	25 (80.6%)	
Extranodal sites				0.382
0-1	88 (84.6%)	60 (82.2%)	28 (90.3%)	
≥2	16 (15.4%)	13 (17.8%)	3 (9.7%)	
IPI				0.561
0	8 (7.7%)	6 (8.2%)	2 (6.5%)	
1	25 (24.0%)	14 (19.2%)	11 (35.5%)	
2	36 (34.6%)	25 (34.2%)	11 (35.5%)	
3	28 (26.9%)	22 (30.1%)	6 (19.4%)	
4	5 (4.8%)	4 (5.5%)	1 (3.2%)	
5	2 (1.9%)	2 (2.7%)	0	
Histopathology				<0.001
PTCL-NOS	47 (45.2%)	40 (54.8%)	7 (22.6%)	
AITL	26 (25.0%)	10 (13.7%)	16 (51.6%)	
Others	31 (29.8%)	23 (31.5%)	8 (25.8%)	
EBER				0.500
Positive	37 (35.6%)	24 (32.9%)	13 (41.9%)	
Negative	64 (61.5%)	46 (63.0%)	18 (58.1%)	
Unknown	3 (2.9%)	3 (4.1%)	0	

PTCL-NOS, peripheral T-cell lymphoma, not otherwise specified; AITL, angioimmunoblastic T-cell lymphoma; ChT, chemotherapy; LDH, lactate dehydrogenase; IPI, international prognostic index.

### Outcomes of Treatment

**Figure 2:**
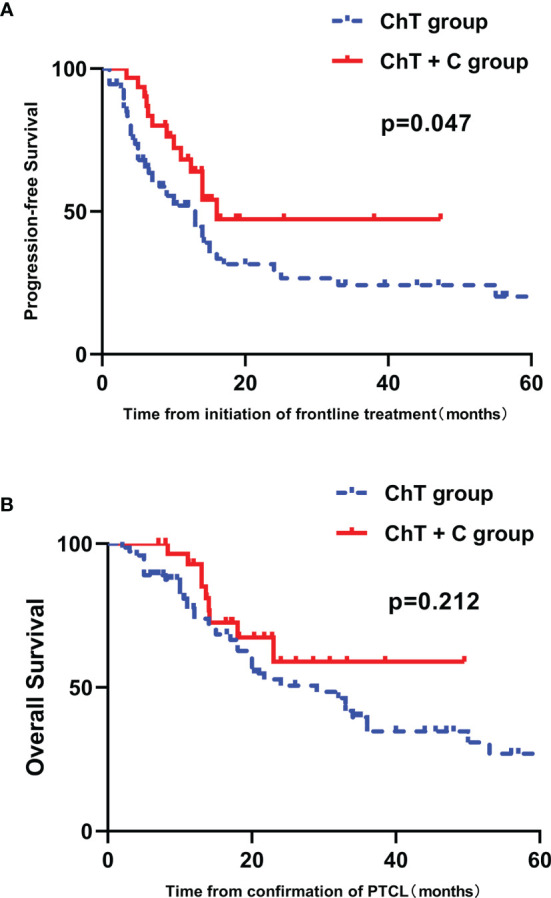
Survival outcomes in patients with untreated PTCL stratified by type of therapy. **(A)** PFS curves in patients stratified by treatment group. **(B)** OS curves in patients stratified by treatment group.

The ORR for ChT group and ChT+C group were 72.6% and 77.4%, respectively (*p*=0.608), with the CR rate of 47.9% and 54.8% (*p*=0.520). Among patients achieving CR/PR in both two groups, the median time to best response was 1.5 months. After the median follow-up of 16.8 months (range 2.0-132.0 months), the median duration of response (CR+PR) in ChT group and ChT+C group were 10.0 months and 14.0 months, with no significant difference (*p*=0.135). The median PFS was 8.5 months (range 1.0-84.0 months) and 12.4 months (range 3.4-47.3 months) in ChT group and ChT+C group, respectively. The median OS were 16.5 months (range 2.0-132.0 months) versus 17.1 months (range 8.0-48.5 months), for ChT and ChT+C group, respectively. The one-year PFS rate and OS rate were 44.6%, and 67.7% in ChT group, respectively, whereas 54.1% and 85.1% in ChT+C group, respectively. Compared with patients who received ChT, those who received ChT+C displayed significantly longer PFS (*p*=0.047, **Figure 2A**). However, there was no significantly statistical difference observed between the two groups in OS (*p*=0.212, **Figure 2B**).

### Prognostic Factor Analysis

**Figure 3:**
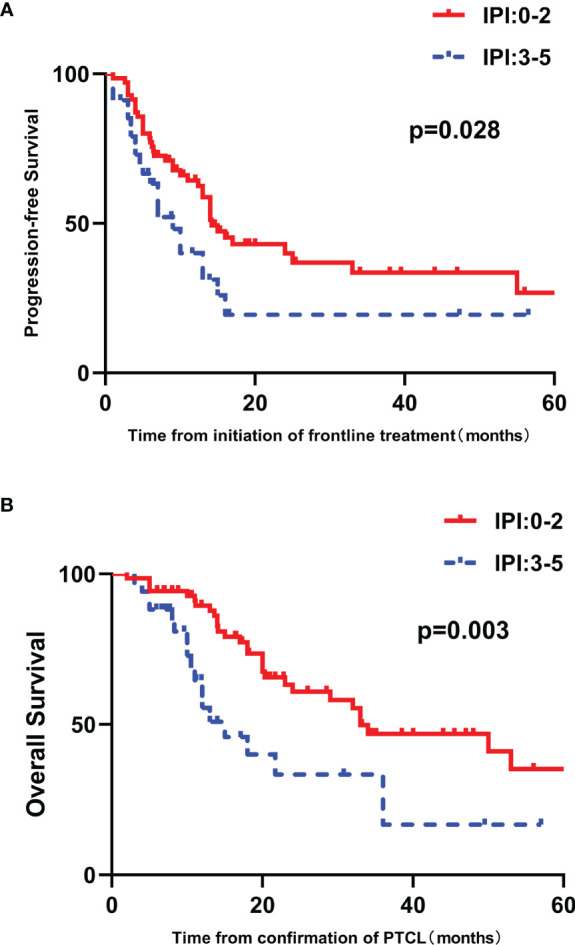
Survival outcomes in patients with untreated PTCL stratified by IPI score. **(A)** PFS curves in patients stratified by IPI score. **(B)** OS curves in patients stratified by IPI score.

**Figure 4:**
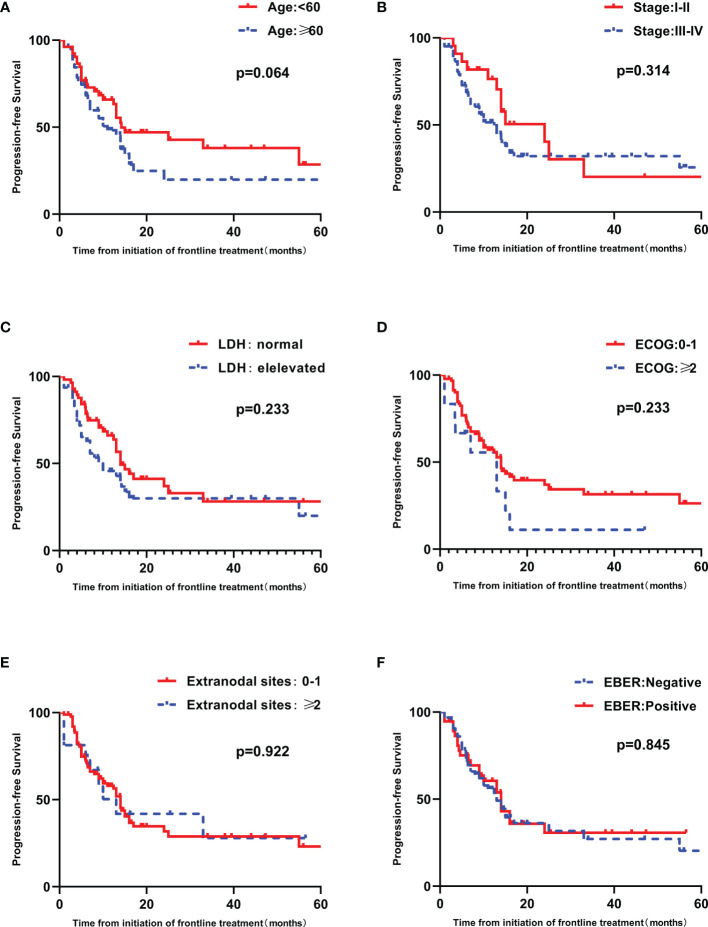
Univariate analyses of PFS in patients with untreated PTCL. **(A)** PFS curves in patients stratified by age. **(B)** PFS curves in patients stratified by Ann Arbor stage. **(C)** PFS curves in patients stratified by LDH. **(D)** PFS curves in patients stratified by ECOG. **(E)** PFS curves in patients stratified by extranodal sites. **(F)** PFS curves in patients stratified by EBER.

High IPI scores have a negative relationship with PFS and OS (*p*=0.028 and 0.003, respectively) (**Figures 3A, B**). The prognostic significance of EBER and the components of the IPI, including age, ECOG performance status, LDH, Ann Arbor stage and number of extranodal sites, were analyzed by univariate analysis. Patients older than sixty years tended to have inferior PFS and OS (*p* = 0.064 and 0.060, respectively) (**Figure 4** and **Supplementary Figure 1**). Patients with poor performance status tended to have a worse overall survival (*p*=0.053, **Supplementary Figure 1**).

Multivariate analysis was done by Cox’s proportional hazards regression model, including frontline treatment regimen and prognostic factors identified by above univariate survival analysis. Frontline treatment regimen was an independent prognostic factor affecting PFS, with the HR of 1.881 (95%CI 1.015 to 3.487, *p*=0.045).

### Subgroup Analysis

**Figure 5:**
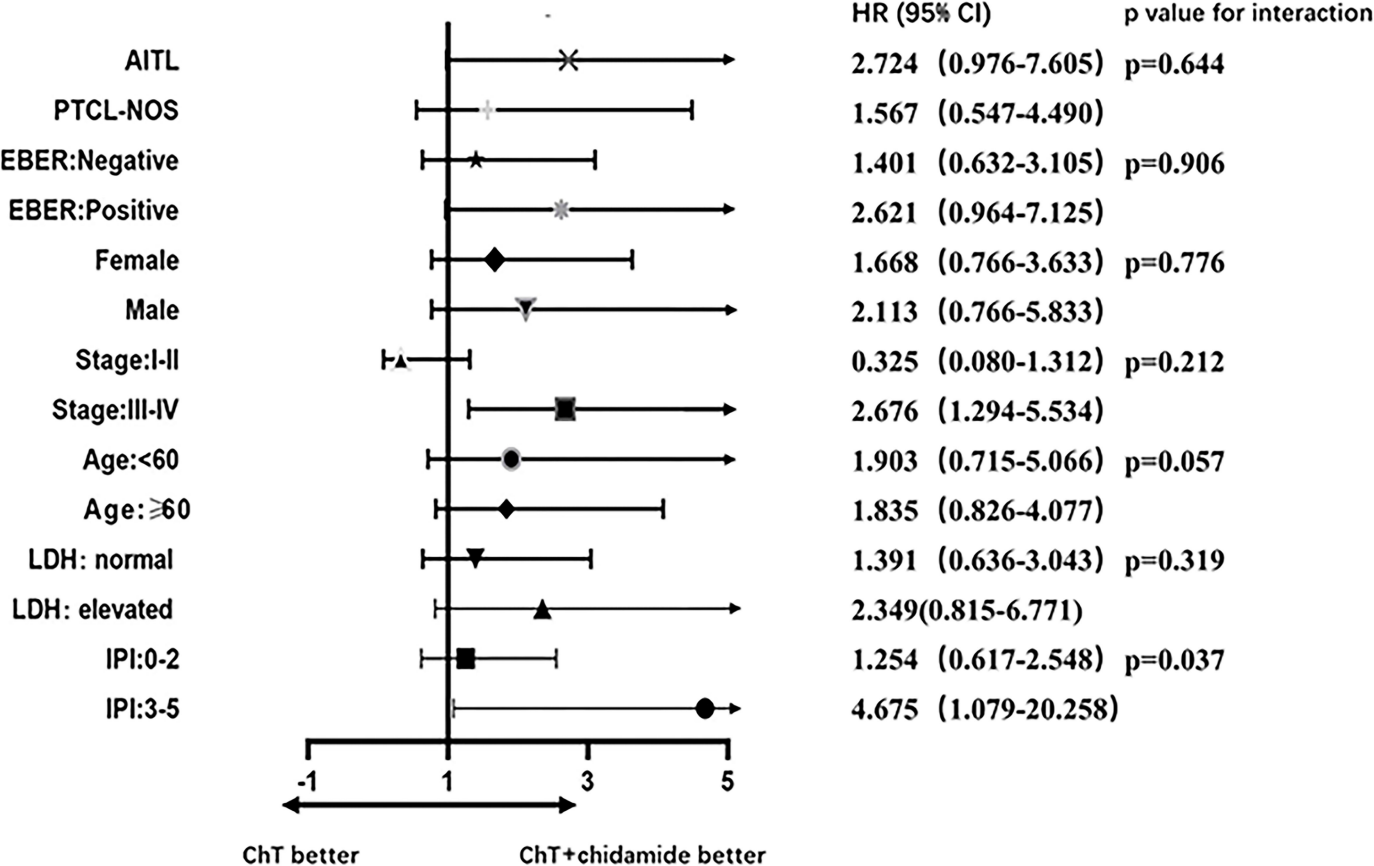
Prespecified subgroup analyses of patients with untreated PTCL.

A forest plot of HRs illustrating the subgroup analyses was shown in **Figure 5**. For patients with high IPI scores (3-5), the HR value for PFS comparing ChT with ChT+C was 4.675 (95%CI 1.079 to 20.258). For patients with advanced disease, the HR value was 2.676 (95%CI 1.294 to 5.534). A test of interaction between IPI and frontline treatment regimen was statistically significant (*p* = 0.037), indicating longer PFS for patients treated with ChT+C in the high-IPI subgroup. There was no evidence of heterogeneity in the effect of chidamide in subgroups defined according to histopathology, EBER, gender, stage, age and LDH.

### Case-Control Analysis

**Figure 6:**
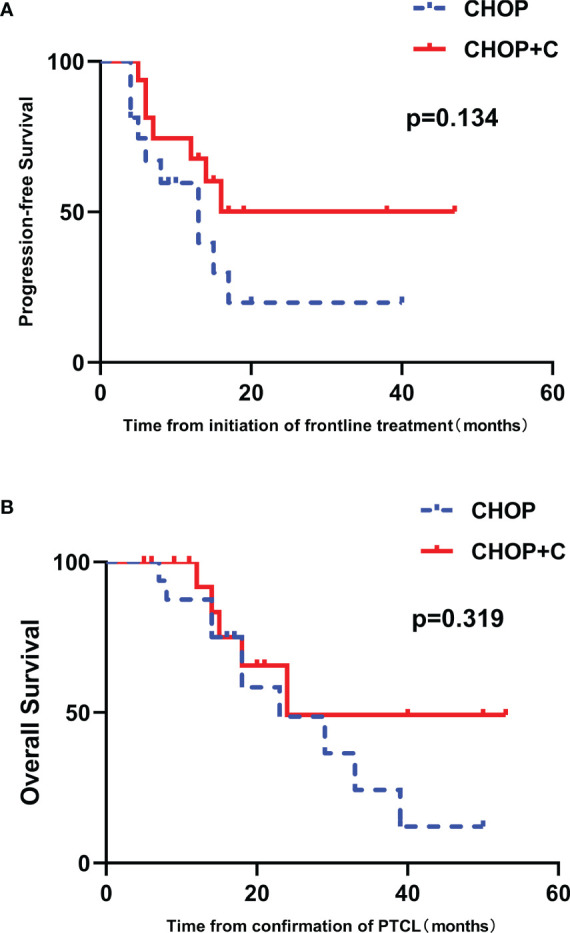
The 1:1 matched case-control analysis of patients with untreated PTCL. **(A)** PFS curves in patients stratified by treatment group. **(B)** OS curves in patients stratified by treatment group.

After matching, 9 female patients (56.3%) and 7 female patients (43.7%) were enrolled in CHOP and CHOP+C group, respectively. Patients with high IPI scores (3-5) and low IPI scores (0-2) accounted for 18.8% (3/16) and 81.2% (13/16) in the two groups, respectively. No statistical difference was found in baseline characteristics between these two groups (*p*>0.05, **Supplementary Table 1**). After a median follow-up of 18.0 months (range 5.0-53.0 months), median PFS was 8.5 months (range, 4.0-40.0 months) in CHOP group versus 14.5 months (range 5.0-47.0 months) in CHOP+C group. The median OS for patients treated with CHOP and CHOP+C was 17.5 months (range 5.0-53.0 months) and 18.0 months (range 7.0-50.0 months), respectively. Median PFS in CHOP+C group was obviously longer compared with CHOP group, but no significant difference was observed in PFS and OS (*p* = 0.134 and 0.319, respectively) (**Figures 6A, B****)**.

### Toxicity Profiles

Hematological adverse events (AEs) were the most frequent treatment-related AEs. In the ChT and ChT+C group, the incidence of grade 3/4 anemia was 17.8% and 16.1% (*p*=0.836), the incidence of grade 3/4 leukocytopenia was 24.7% and 17.6% (*p*=0.557), and the incidence of grade 3/4 thrombocytopenia was 23.3%, and 16.1% patients (*p*=0.414), respectively. Frequency of AEs did not appear to be significantly different between the two groups. None of the adverse reactions resulted to death.

## Discussion

In this study, we examined whether adding chidamide to traditional chemotherapy can potentiate the antitumor activities and improve survival, in the frontline setting of PTCL. Compared with patients treated with ChT, those treated with ChT+C group superior PFS. However, there was no significantly statistical difference observed between the two groups in OS. High IPI scores were associated with poor PFS and OS, consistent with previous data (13). Among patient with untreated PTCL, frontline treatment regimen was considered as an independent prognostic factor for PFS. Severe treatment-related AEs were infrequent. The incidence of hematological toxicity is highest in both two groups, without significant difference. The results of our study indicated the combination of ChT and chidamide confers better prognosis to untreated PTCL patients with acceptable toxicity.

In the era of traditional chemotherapy, a large proportion of PTCL patients suffered frequent relapse and unfavorable outcome (14). Chidamide, as a kind of HACDi, may bring us a promising future. HDACis promote apoptosis and inhibit angiogenesis through acetylation of both histone and non-histone proteins (15, 16). However, HDACi monotherapy demonstrated modest and short-lived effectiveness, without complete and durable responses (10). Thus, in order to improve the prognosis, more optimal combination therapeutic approach for PTCL should be optimized further. In addition, previous researches have proven that the synergy between HDAC inhibitors and cytotoxic agents *in vitro* (17–19). Given the potential synergistic effect, for patients with untreated PTCL, it is reasonable to explore the integration of chidamide into standard chemotherapy.

Subgroup analyses revealed that remarkable differences in PFS among subgroups as defined by IPI. Within the ChT+C group, more PFS benefit was observed in the high-IPI subgroup. In contrast, chidamide had no noticeable impact on PFS in the low-IPI subgroup. A nominally significant interaction between IPI and frontline treatment was observed, which indicates IPI as a prognostic indicator should be taken into consideration in clinical settings. Based on the results of our study, IPI was an independent risk factor for poor prognosis. Therefore, one reasonable explanation for PFS benefit in the subgroup of patients with high IPI scores is that risk factor can be overcome by chidamide to a certain extent. Patients with low IPI scores tended to have better survival outcome and derive less benefit from chidamide, so ChT alone might be an appropriate treatment option for this cohort. These findings suggested that baseline characteristics of patients could guide individualized treatment selection.

According to the survival analysis in our study, IPI was found to have prognostic significance in the survival of the patients with newly diagnosed PTCL, which are consistent with previous studies. Additionally, the choice of chemotherapy regimen may make a difference to the survival outcome. There was the potential baseline imbalance in IPI and chemotherapy regimen between the two groups. Hence, a 1:1 (low IPI scores: high IPI scores) matched case-control analysis was conducted, selecting IPI as the matching conditions and using the propensity score matching method in the patients receiving CHOP or CHOP+C. The results indicated that median PFS in CHOP+C group was obviously longer compared with CHOP group, but there was no significant difference observed. Given that most patients included in case-control analysis had low IPI scores (81.2%), the non-significantly different survival outcome may be attributed to the less benefit of ChT+C in patients with low IPI, which was consistent with the subgroup analysis.

The toxicity profile was generally featured with the predictable and manageable hematological events. The AEs following frontline treatment was analogous, regardless of whether chidamide was utilized.

In fact, there were several restrictions in our study. Firstly, there is growing evidence that ASCT as the frontline consolidation therapy can improve survival (6). However, most of patients included in this study did not undergo ASCT because of their inadequate understanding of ASCT and poor economic conditions. Secondly, due to the lack of an effective treatment regimen, physician and patient preferences as confounding variables might have influenced the outcome. Thirdly, this observational study with small sample size has a limitation towards conducting further analysis. Taken together, studies with larger randomized design are necessary to validate our findings.

## Conclusions

To sum up, traditional chemotherapy combined with chidamide may prolong the PFS in patients with newly diagnosed PTCL, especially for patients with high IPI scores. Treatment-related toxicities characterized by hematological events were manageable and reversible. It is attractive to explore further with a larger and randomized study in order to identify the more excellent strategy.

## Data Availability Statement

The raw data supporting the conclusions of this article will be made available by the authors, without undue reservation.

## Ethics Statement

The studies involving human participants were reviewed and approved by the review board of Sun Yat-sen University Cancer Center. The patients/participants provided their written informed consent to participate in this study.

## Author Contributions

All authors contributed to the study conception and design. Material preparation, data collection and analysis were performed by JW and NS. The first draft of the manuscript was written by JW, and all authors commented on previous versions of the manuscript. All authors read and approved the final manuscript.

## Funding

This study was supported by grants from the Special Support Program of Sun Yat-sen University Cancer Center (PT19020401, QC), the Science and Technology Planning Project of Guangzhou, China (202002030205, QC), and the Clinical Oncology Foundation of Chinese Society of Clinical Oncology (Y-XD2019-124, QC). The open access publication fees were paid by the Science and Technology Planning Project of Guangzhou, China (202002030205, QC).

## Conflict of Interest

The authors declare that the research was conducted in the absence of any commercial or financial relationships that could be construed as a potential conflict of interest.

The handling editor declared a past co-authorship with the authors NS, SM, XT, HuH, and QC.

## Publisher’s Note

All claims expressed in this article are solely those of the authors and do not necessarily represent those of their affiliated organizations, or those of the publisher, the editors and the reviewers. Any product that may be evaluated in this article, or claim that may be made by its manufacturer, is not guaranteed or endorsed by the publisher.

## Supplementary Material

Supplementary Figure 1 Univariate analyses of OS in patients with untreated PTCL. **(A)** OS curves in patients stratified by age. **(B)** OS curves in patients stratified by Ann Arbor stage. **(C)** OS curves in patients stratified by LDH. **(D)** OS curves in patients stratified by ECOG. **(E)** OS curves in patients stratified by extranodal sites. **(F)** OS curves in patients stratified by EBER.
