# Supplementary figures and images for: Comparison of Chemotherapy Combined With Chidamide Versus Chemotherapy in the Frontline Treatment for Peripheral T-Cell Lymphoma
Source: Front Immunol. 2022 Feb 2;13:835103. doi: 10.3389/fimmu.2022.835103 (PMC8847145; doi:10.3389/fimmu.2022.835103)

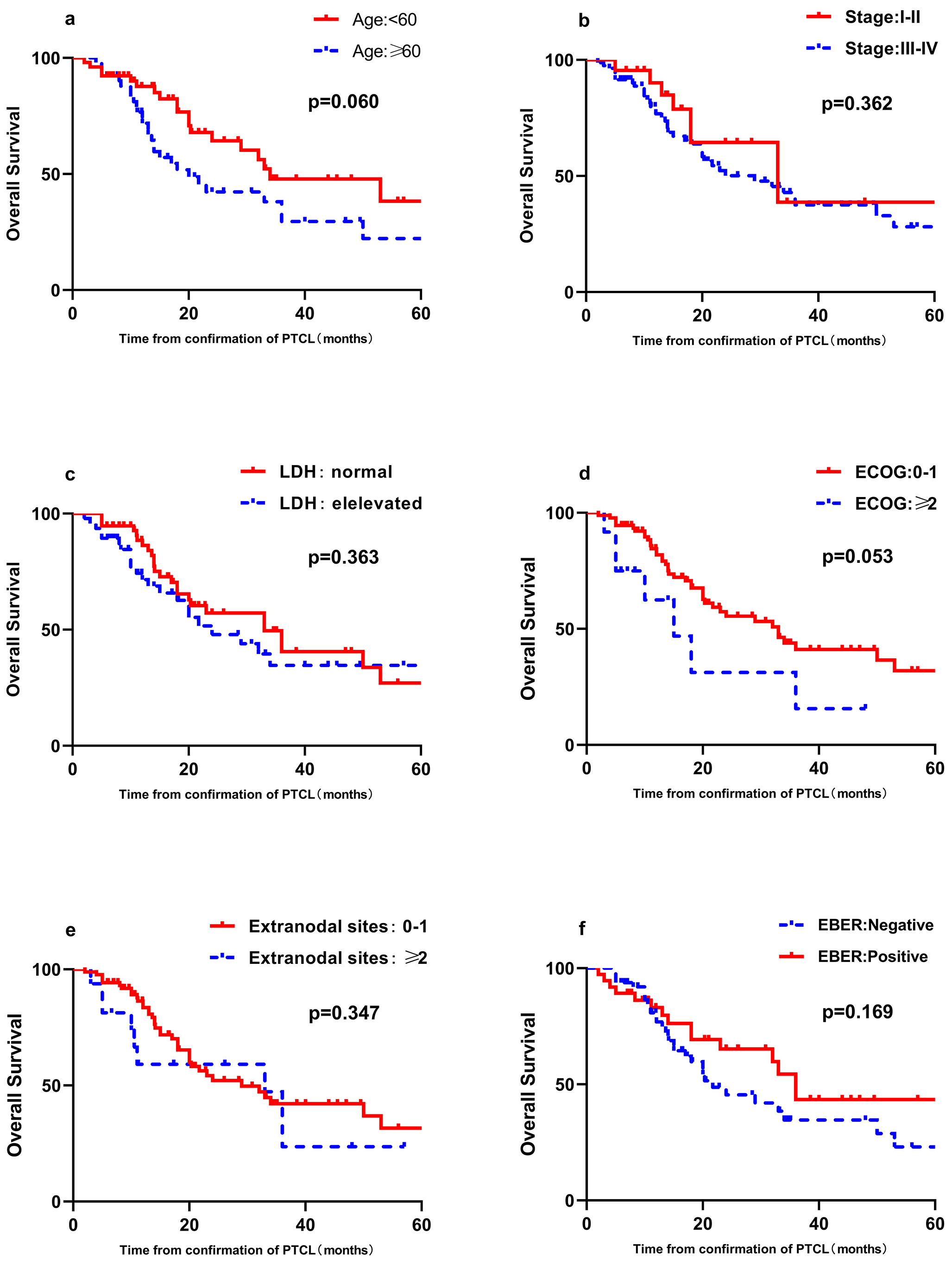

Supplement: Supplementary Figure 1 — Univariate analyses of OS in patients with untreated PTCL. (A) OS curves in patients stratified by age. (B) OS curves in patients stratified by Ann Arbor stage. (C) OS curves in patients stratified by LDH. (D) OS curves in patients stratified by ECOG. (E) OS curves in patients stratified by extranodal sites. (F) OS curves in patients stratified by EBER. [file Image_1.tif]
