# Supplementary material for: Comparison of Chemotherapy Combined With Chidamide Versus Chemotherapy in the Frontline Treatment for Peripheral T-Cell Lymphoma
Source: Front Immunol. 2022 Feb 2;13:835103. doi: 10.3389/fimmu.2022.835103 (PMC8847145; doi:10.3389/fimmu.2022.835103)
Supplement: Supplementary file 2 [file Table_1.docx]

**Supplementary table1. Baseline characteristics of 32 matched patients with PTCL**

| **Characteristics** | **Overall**  **n=32** | **ChT**  **n=16** | **ChT+C**  **n=16** | ***p*** |
| --- | --- | --- | --- | --- |
| **Age, years** |  |  |  | **0.593** |
| **median** | **56.0** | **57.5** | **53.5** |  |
| **range** | **(27-78)** | **(31-75)** | **(27-78)** |  |
| **Gender** |  |  |  | **1.000** |
| **Male** | **18(56.3%)** | **9(56.3%)** | **9(56.3%)** |  |
| **Female** | **14(43.7%)** | **7(43.7%)** | **7(43.7%)** |  |
| **LDH** |  |  |  | **0.710** |
| **Normal** | **21(65.6%)** | **11(68.8%)** | **10(62.5%)** |  |
| **Elevated** | **11(34.4%)** | **5(31.2%)** | **6(37.5%)** |  |
| **ECOG** |  |  |  | **1.000** |
| **0-1** | **31(96.9%)** | **16(100%)** | **15(93.8%)** |  |
| **≥2** | **1(3.1%)** | **0** | **1(6.2%)** |  |
| **Stage** |  |  |  | **0.654** |
| **I-II** | **6(18.8%)** | **2(12.5%)** | **4(25.0%)** |  |
| **III-IV** | **26(81.2%)** | **14(87.5%)** | **12(75.0%)** |  |
| **Extranodal** **sites** |  |  |  | **1.000** |
| **0-1** | **30(93.8%)** | **15(93.8%)** | **15(93.8%)** |  |
| **≥2** | **2(6.2%)** | **1(6.2%)** | **1(6.2%)** |  |
| **Histopathology** |  |  |  | **0.453** |
| **PTCL-NOS** | **16(50.0%)** | **10(62.5%)** | **6(37.5%)** |  |
| **AITL** | **6(18.8%)** | **2(12.5%)** | **4(25.0%)** |  |
| **Others** | **10(31.2%)** | **4(25.0%)** | **6(37.5%)** |  |
| **EBER** |  |  |  | **1.000** |
| **Positive** | **9(35.6%)** | **4(25.0%)** | **5(31.3%)** |  |
| **Negative** | **23(61.5%)** | **12(75.0%)** | **11(68.7%)** |  |
|  |  |  |  |  |

**PTCL-NOS: peripheral T-cell lymphoma, not otherwise specified; AITL: angioimmunoblastic T-cell lymphoma; ChT: chemotherapy; LDH: lactate dehydrogenase**
